# Differential Association of Metabolic Risk Factors with Open Angle Glaucoma according to Obesity in a Korean Population

**DOI:** 10.1038/srep38283

**Published:** 2016-12-22

**Authors:** Hyun-Ah Kim, Kyungdo Han, Yun-Ah Lee, Jin A Choi, Yong-Moon Park

**Affiliations:** 1 Department of Ophthalmology, College of Medicine, The Catholic University of Korea, Seoul, Korea; 2 Department of Biostatistics, College of Medicine, The Catholic University of Korea, Seoul, Korea; 3 Department of Family Medicine, St. Vincent’s Hospital, College of Medicine, The Catholic University of Korea, Seoul, Korea; 4 Department of Ophthalmology, St. Vincent’s Hospital, College of Medicine, The Catholic University of Korea, Seoul, Korea; 5 Epidemiology Branch, National Institute of Environmental Health Sciences, National Institutes of Health, Research Triangle Park, NC, USA

## Abstract

The associations of the metabolic syndrome (MetS) with intraocular pressure and primary open angle glaucoma (OAG) have been reported. This study aimed to determine whether a difference in association exists between OAG and metabolic risk factors according to obesity status among Korean adults. A total of 8,816 participants (≥40 years) in the Korea National Health and Nutrition Examination Survey were classified into obese, body mass index (BMI) ≥ 25 kg/m^2^ and non-obese, BMI < 25 kg/m^2^. The prevalence of MetS was 40.1% in non-obese OAG and 66.0% in obese OAG. The prevalence of OAG increased with increasing number components for MetS in total population and in non-obese subjects (P < 0.001, respectively), while the prevalence of OAG was not associated with number of components for MetS in obese subjects (P = 0.14). In non-obese individuals, subjects with high triglycerides, high blood pressure (BP), and MetS were more likely to have OAG compared with those without high triglycerides, high BP, and MetS after adjusting for potential confounders. However, MetS or its components exhibited no significant association with glaucoma status in obese individuals. Our study provides understanding on the differences in association of OAG with MetS and its components according to obesity status.

Glaucoma is the second leading cause of global blindness following cataract and accounts for approximately 8% of blindness cases^1^. Among subtypes of glaucoma, open angle glaucoma (OAG) is reported to be the most common^2^,^3^,^4^; more specifically, normal tension glaucoma (NTG) predominates primary open angle glaucoma (POAG) in Asians. NTG was reported to comprise about 77% of OAG in Asians and is characterized by normal baseline intraocular pressure (IOP) and slow progression^5^, is asymptomatic until late stages, and is therefore difficult to detect if screening examinations are not performed.

Of the many risk factors of OAG, IOP is considered as a predominant risk factor worldwide. Recently, associations between IOP and components of metabolic syndrome (MetS) have been reported in both genders in diverse ethnic groups^6^,^7^,^8^. Moreover, associations between OAG and components of MetS have also been reported^9^. Components such as blood pressure (BP) showed differential association with OAG according to the level of BP, with low diastolic and high systolic BP being associated with OAG^10^,^11^. Increasing evidence suggests strong association between OAG and MetS^12^,^13^. Like many other metabolic diseases, the relationship with obesity has been previously evaluated, and numerous studies reported that lower body mass index (BMI) is associated with increased risk of OAG^14^,^15^.

Obesity, a growing public health problem especially in Asian countries^16^,^17^, is thought to cause metabolic abnormalities, such as insulin resistance, dyslipidemia, hypertension, which are risk factors for cardiovascular disease and type II diabetes^18^. However, there has been recent interest in the heterogeneity of cardiovascular disease risk within the traditional body mass index (BMI) categories: A substantial proportion of overweight or obese adults are metabolically healthy, whereas a considerable proportion of normal-weight adults exhibit a clustering of metabolic abnormalities^19^,^20^. This subgroup of metabolically abnormal but non-obese individuals are reported to be associated with high risk of cardiovascular disease^19^,^21^,^22^. It has been commonly reported that despite lower BMI, Asians tend to have higher percent body fat and are more insulin resistant than Caucasians^23^,^24^, with relatively high prevalence of metabolically obese but normal weight population^16^,^25^.

Considering that OAG is affected by hemodynamic risk factors^9^,^10^,^11^, the disease may also show differential association with obesity and MetS. Despite the possible clinical significance, the associations between OAG and metabolic risk factors according to obesity status have not been addressed in large population-based studies. Therefore, the present study was conducted to investigate the associations between metabolic risk factors and OAG in Korean adults aged 40 years or older and stratified by obesity status, focusing on possible differences in these associations.

## Results

The prevalence of OAG was 3.90% overall, 4.22% among non-obese participants, and 3.25% among obese participants. The study population included 210 underweight (BMI < 18.5 kg/m^2^), 3,203 normal weight (18.5 ≤ BMI < 23 kg/m^2^), 2,324 overweight (23 ≤ BMI < 25 kg/m^2^), and 3,079 obese (BMI ≥ 25 kg/m^2^) participants, who were grouped according to the recommendations for Asians by the WHO Pacific Regional Office^26^,^27^. The general and clinical characteristics of the subjects are shown according to presence of glaucoma and obesity in Table 1. In both non-obese and obese groups, age, IOP, household income, education level, diagnosis of hypertension and systolic BP were significantly different between non-glaucoma and glaucoma subjects. Spherical equivalent, TGs, HDL-cholesterol, and diagnosis of MetS in the non-obese group were also significantly different between non-glaucoma and glaucoma subjects.

In total population, the diagnosis of hypertension, the prevalence of high FPG, the prevalence of high TGs, and the presence of MetS were significantly higher in glaucoma subjects compared with non-glaucoma subjects (P < 0.001, 0.043, 0.002, and <0.001, respectively, Table 2). However, contrasting results on diagnosis of MetS and its components were found in non-glaucoma and glaucoma subjects according to obesity status.

In the non-obese group, the diagnosis of hypertension, prevalence of high TGs, prevalence of low HDL-cholesterol, and presence of MetS were significantly higher in glaucoma subjects compared with non-glaucoma subjects (*P* < 0.001, <0.001, 0.009, and <0.001, respectively) However, among obese individuals, only the diagnosis of hypertension was significantly higher in glaucoma subjects than non-glaucoma subjects (*P* = 0.017).

Table 3 shows that, in total population, subjects with hypertension, high TGs, and MetS were significantly more likely to have glaucoma after adjusting for surveyed year, age, and sex (Model 1, odds ratio (OR), 1.58; 95% confidence interval (CI), 1.13–2.21; OR, 1.43; 95% CI, 1.08–1.90; OR, 1.37; 95% CI, 1.03–1.83, respectively), which still remained significant after additional adjustment for IOP, household income, exercise, education level, smoking status, alcohol consumption, BMI, and dyslipidemia medication use (Model 2). Similarly, in non-obese individuals, subjects with hypertension, high TGs, and MetS were also significantly more likely to have glaucoma (Model 2, OR, 1.88; 95% CI, 1.29–2.74; OR, 1.65, 95% CI, 1.12–2.43; OR, 2.00, 95% CI, 1.34–3.00, respectively). Unlike non-obese individuals, MetS components in obese individuals exhibited no statistically significant association with glaucoma.

**Figure 1:**
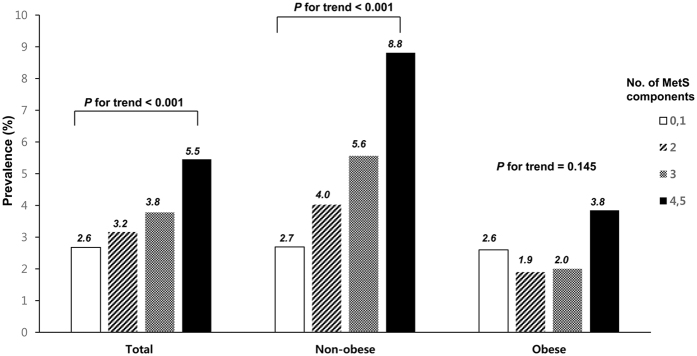
Prevalence of glaucoma according to the number of MetS components in total study participant, non-obese and obese participants. Among non-obese participants, the prevalence of glaucoma increased with increasing number of MetS components (*p* for trend <0.001), while the prevalence of glaucoma was not associated with the number of MetS components among obese individuals (*p* for trend = 0.145).

The prevalence of glaucoma according to the number of MetS components in non-obese and obese individuals is presented in Fig. 1. The prevalence of glaucoma significantly increased with increasing number of components for MetS among total population and non-obese subjects (*p*-value for trend <0.001 and <0.001), while the prevalence of glaucoma was not significantly associated with the number of components for MetS among obese subjects (*p*-value for trend = 0.145).

## Discussion

To the best of our knowledge, this is the first large, population-based study to examine the associations between metabolic risk factors and OAG according to obesity status among an Asian adult population. In this cross-sectional study comprising Korean adults aged 40 years or older who participated in KNHANES during the years 2010–2012, we found a positive association between elevated blood pressure, high TGs, and MetS and OAG among the non-obese population after adjusting for potential confounders (Table 3). Moreover, the prevalence of OAG was positively associated with the number of MetS components in the non-obese-population, whereas no significant association was observed in the obese population (Fig. 1).

The obese population in our study not only showed no association between OAG and MetS, but also had a lower prevalence of OAG itself than the non-obese population. This is in agreement with previous studies which revealed that higher BMI is associated with lower risk of OAG among Asians^15^ as well as Caucasians^14^. In our study, the prevalence of OAG was 4.22% among non-obese subjects, 3.25% among obese subjects, and 3.90% in the total study population. This is similar to the prevalence published in other epidemiologic studies in Korea (Namil study, 3.5%)^3^ and Japan (Tajimi study, 3.9%)^4^.

Although the exact mechanism underlying this phenomenon is not known, a few factors may have played a role in the protective effect against OAG among the obese population, effective enough to overcome the adverse influence of MetS and it’s components on the development of OAG. First, leptin is primarily synthesized in adipose tissue, and in addition to its well-known action on the hypothalamus for appetite and metabolism control, neuroprotective activity in the brain^28^ has also been reported. The degeneration and progressive loss of retinal ganglion cells are pathophysiological features of glaucoma^29^. Leptin receptors were found on axons of such retinal ganglion cells^30^, with leptin acting as a neuroprotective agent in glaucoma^31^. Plasma leptin level is known to increase proportionally with an increase in adipose tissue^32^. Second, estrogen, which is increased in both men and women in the obese population^33^, has a neuroprotective effect through estrogen receptors in retinal ganglion cells^34^ as well as increasing ocular blood flow^35^. Prior studies using animal models revealed retinal protective effect of 17-beta-estradiol on retinal ganglion cells^34^, and decreased retinal nerve fiber thickness was found in postmenopausal women not on hormone replacement therapy compared with those who did^36^. In addition, increased neuropeptide Y release which is known to be associated with obesity^37^ has been reported to inhibit the decrease in the number of ganglion cells^38^. Animal models showed neuroprotective role of neuropeptide Y against necrotic and apoptotic retinal ganglion cell death induced by glutamate^38^, which is the main excitatory neurotransmitter in the retina^39^.

In our study, individuals with high TGs were significantly more likely to have OAG in the non-obese and total population (Table 3). This result is consistent with a previous nationwide cohort study carried out in Asia which reported higher prevalence of high TGs among OAG patients^40^. Since dyslipidemia is a well-established risk factor for atherosclerosis^41^, high TGs may contribute to OAG development through decreased retinal and choroidal perfusion and ischemic damage of the retinal ganglion cells. In the same context, the use of statins is reported to have a neuroprotective effect against OAG through improved perfusion, as demonstrated in an ischemia-reperfusion animal model^42^. Although the definition of high TGs included individuals on treatment for high TGs in the present study, high TGs in the non-obese and total population were still strongly associated with OAG even after additional adjustment for dyslipidemia medication use in Model 2 of Table 3. In contrast, high TGs in the obese population were not associated with increased prevalence of OAG.

Low HDL cholesterol was not associated with increased prevalence of OAG in all three groups after adjustment for confounding variables including medication use for dyslipidemia (Table 3). Similarly, in other studies conducted in Asia, which did not divide the study groups according to obesity, no association was found between low HDL cholesterol and OAG^43^,^44^. Among the young Korean population aged between 10 and 39 years with normal baseline IOP, low HDL cholesterol has been identified as a significant variable increasing the risk of OAG, suggesting a possible difference in the association in younger age groups^6^.

In the present analysis, non-obese individuals with hypertension were more likely to be associated with OAG than were those without hypertension after adjustment for confounding variables such as IOP and BMI, while no such association was observed among obese individuals. The association between high systolic BP and increased prevalence of OAG has been verified in many previous studies^10^,^11^, as high systolic BP can result in low perfusion pressure due to reduced vessel diameter in the short-term and arteriosclerosis in the long-term^45^. Systemic use of beta-blocker and nitrate to lower blood pressure has been previously addressed to be associated with lower IOP^27^, possibly providing a protective effect against glaucoma. In this study, we further found that individuals with high BP or those on antihypertensive medications were associated with higher prevalence of OAG, independent of IOP.

High prevalence of myopia is one of the well-known ocular characteristics of East Asians, with a prevalence of 35.7% among Korean adults over 40 years of age, which is higher than Western Europe and United States, and similar to China and Japan^46^. Despite the well-established association between myopia and OAG^47^, when divided into groups according to obesity status, OAG subjects were significantly more myopic than non-OAG subjects only in the non-obese group, and not in the obese group. Moreover, although smoking is one of the recognized risk factors of glaucoma^48^, there was no difference in smoking status between non-OAG and OAG subjects when stratified by obesity status.

OAG in the present study included both POAG with high IOP and NTG, as IOP was not taken into account in the diagnosis. In addition to the well-known relationship between IOP and glaucoma, the association between IOP and MetS has also been previously established in both men^6^ and women^7^ of various ethnic groups^8^. However, our study is interesting in that the relationship between OAG and MetS was evaluated according to obesity, independent of IOP.

Our study should be interpreted with consideration of the following limitations. First, causality cannot be determined because this was a cross-sectional analysis. Second, instead of separately studying the effect of statin use on OAG, subjects with statin use were defined as having dyslipidemia in the diagnosis of MetS components. However, despite the reported protective effect of statin use in glaucoma^49^, subjects with high TGs were more likely to be associated with OAG in the non-obese group and all study participants even after further adjusting for dyslipidemia medication use, implying that the association is powerful enough to overcome the protective effect of statins. Third, MetS components were defined using single lipid and glucose measurement, which may have biased the results. Fourth, the relationship between OAG and metabolic risk factors were not separately evaluated for male and female, despite the fact that sex hormones may play a role in the pathogenesis of OAG. Further investigations on the association between OAG and metabolic syndrome components in the non-obese population according to gender may yield additional interesting results. Fifth, we included both POAG and NTG in the analysis. Although adjustment for IOP was carried out to minimize the effect of confounding, since NTG is much more prevalent among East Asians^3^,^4^, a separate analysis may also provide interesting findings. Finally, OAG was diagnosed based on FDT results instead of using the standard automated perimetry (SAP). This may be responsible for slightly higher prevalence of OAG in this study (3.9%) compared with other epidemiologic study in Korea using SAP (3.5%)^3^, as FDT has a higher false positive rate than SAP^17^. Despite these limitations, the present study was based on a nationally representative sample of the Korean adult population, which is a major strength. Furthermore, it is the first large population-based study to examine the associations between OAG and components of MetS according to obesity status.

In conclusion, high TGs, high BP, and MetS were independently associated with increased prevalence of OAG in non-obese individuals, while such associations were not found in obese individuals. Moreover, the prevalence of OAG increased with increasing number of MetS components among the non-obese population, whereas such trend was not observed among the obese population. This indicates a difference in association of components of MetS and glaucoma according to obesity status, and highlights a possible need for special attention to metabolically obese but normal weight adults, especially those with high BP and high TGs, on screening and detecting glaucoma.

## Patients and Methods

### Study Population

This study was conducted using data from the Korea National Health and Nutrition Examination Survey V (KNHANES) 2010–2012. KNHANES is a cross-sectional, nationally representative sample of the non-institutionalized Korean civilian population aged one year or older and employs a rolling sampling design that implements a complex, stratified, multistage probability sample design. KNHANES is organized regularly by the Korean Ministry of Health and Welfare to monitor the health and nutritional status of people in South Korea^50^,^51^. It is comprised of a health interview survey, health examination survey, and nutrition survey. The ophthalmologic survey was first introduced in KNHANES in 2008 and involves ophthalmologic interviews and examinations for participants aged 3 years or older. In this study, a total of 12,881 individuals aged 40 years or older who completed the ophthalmologic survey were selected. Of these 12,881 subjects, we excluded individuals who had missing data in the health interview survey or health examination survey. After exclusion, a total of 8,816 subjects were selected as the study population. The KNHANES V was conducted according to the Declaration of Helsinki and was carried out by specially trained interviewers or examiners who were not provided with any prior information about the participants, and all participants signed an informed consent form. This study was approved by the Institutional Review Board of the St. Vincent’s hospital, the Catholic University of Korea.

### Measurements

In the health interview survey, trained interviewers recorded information about demographic and health-related characteristics including age, education, residence, and household income. Information about lifestyle characteristics, including smoking, drinking alcohol, and exercise, were recorded by self-reported questionnaires. Residence was categorized as urban (8 major cities in South Korea: Seoul, Gyeonggi, Busan, Daegu, Incheon, Gwangju, Daejeoun, Ulsan) or rural (8 other provinces in South Korea). Household income was divided into quartiles after being adjusted for the number of family members. Participants were grouped in the low income group if their household income was categorized into the lowest quartile. Education level was categorized into four groups by highest education achieved: elementary school, middle school, high school, and university. Subjects were categorized in the high education group if their highest education was high school or university. Smoking status was categorized into three groups: non-smokers, current smokers, and ex-smokers. Alcohol consumption status was also classified into three groups: non-drinker, mild-to-moderate drinker (<30.0 g alcohol/day), and heavy drinker (≥30.0 g alcohol/day). Regular exercise was defined as moderate-intensity physical activity (e.g., carrying a light load, cycling at a regular pace, playing tennis) for at least 20 minutes at a time and at least three times a week.

Height was measured to the nearest 0.1 cm using a portable stadiometer (SECA 225, SECA Deutschland, Hamburg, Germany) while the participants were standing barefoot. Weight was measured to the nearest 0.1 kg using an electronic scale (GL-6000-20, CAS KOREA, Seoul, Korea) while the participants wore a lightweight gown. BMI was calculated as weight in kilograms divided by height in meters squared (kg/m^2^). Waist circumference (WC) was measured after normal expiration to the nearest 0.1 cm using a measuring tape (SECA 200, SECA Deutschland) at the mid-level between the lower margin of the ribcage and the iliac crest at the mid-axillary line. BP was measured from the right arm using a standard mercury sphygmomanometer (Baumanometer, WA Baum Co., New York, NY, USA) after 5 minutes of rest in the sitting position. Systolic and diastolic BPs were measured three times at 30 second intervals, and the average of the final two values was used for analysis. Hypertension was defined as systolic BP ≥ 140 mmHg, diastolic BP ≥ 90 mmHg, or use of antihypertensive medications.

Venous blood samples were collected from participants who had been fasting for at least 8 hours. Fasting plasma glucose (FPG), high-density lipoprotein (HDL) cholesterol, total cholesterol, and triglycerides (TGs) were measured using a Hitachi Automatic Analyzer 7600 (Hitachi, Tokyo, Japan). Low-density lipoprotein (LDL) cholesterol was calculated using the Friedewald formula. Diabetes mellitus was defined as FPG ≥ 126 mg/dL or use of insulin or anti-hyperglycemic medications.

All ophthalmologic examinations were performed using a slit-lamp (Haag-Streit model BQ-900; Haag-Streit AG, Koeniz, Switzerland) by ophthalmologists. The participants underwent ophthalmologic interviews, visual acuity measurements, slit-lamp examinations of the anterior segment, and IOP measurements. IOP was measured once per eye with a Goldmann applanation tonometer during slit-lamp examination. A digital non-mydriatic retinal camera and a Nikon D-80 digital camera (Nikon Inc., Tokyo, Japan) were used to obtain fundus images from all participants, without pupil dilation. Visual field testing by frequency-doubling technology (FDT) (Humphrey Matrix; Carl Zeiss Meditec, Inc., Dublin, CA) using the N-30-1 screening test was performed if the participants were suspected of having glaucoma and met any of the following criteria: (1) IOP ≥ 22 mmHg or glaucomatous optic disc; (2) vertical cup-to-disc ratio (VCDR) or horizontal cup-to-disc ratio (HCDR) ≥ 0.5; (3) optic disc hemorrhage; (4) retinal nerve fiber layer defect; or (5) non-adherence to the ISNT rule (i.e., normally, the neuroretinal rim thicknesses are in the following order: inferior > superior > nasal > temporal). FDT was repeated if the rate of fixation errors was >0.33 or if the false-positive rate was >0.33. Only one eye from each subject was used for statistical analysis. The data from the eye diagnosed with OAG were used for analysis, and the worse eye was chosen for analysis in bilateral cases. For control subjects, the right eye was chosen for analysis because the IOPs in the right and left eyes were highly correlated (Pearson’s correlation = 0.79, *P* < 0.001).

### Definition of glaucoma

We defined OAG as “glaucoma” in this study. OAG was diagnosed based on the International Society of Geographical and Epidemiological Ophthalmology Criteria. OAG was defined as the presence of an open angle (peripheral anterior chamber depth >1/4 corneal thickness) and satisfaction of any one of the following category 1 or 2 criteria: Category 1 required both a visual field defect consistent with glaucoma and either a VCDR ≥ 0.7 (97.5th percentile) or a VCDR difference ≥0.2 between eyes (97.5th percentile) [if FDT results are present and have fixation error and false-positive error ≤1]; Category 2 required a VCDR ≥ 0.9 (99.5th percentile) or a VCDR difference ≥0.3 between eyes (99.5^th^ percentile) [if FDT results are absent or if FDT results have fixation error and false-positive error ≥2]. IOP was not taken into consideration when establishing the diagnosis of OAG.

### Definitions of obesity and MetS

In this study, obesity was defined as a BMI ≥ 25 kg/m^2^, as recommended for Asians by the WHO Western Pacific Regional Office^26^,^27^. MetS was diagnosed according to the National Cholesterol Education Program Adult Treatment Panel III’s modified definition of MetS^52^. The WC criteria were modified to be suitable for the Asian population^53^. MetS was diagnosed when more than three of the following risk factors were present: (1) WC ≥ 90 cm for men or WC  ≥80 cm for women; (2) serum TGs ≥ 150 mg/dL or treatment for high TGs; (3) HDL cholesterol <40 mg/dL for men, HDL cholesterol <50 mg/dL for women, or undergoing treatment for HDL cholesterol abnormalities; (4) systolic BP ≥ 130 mmHg, diastolic BP ≥ 85 mmHg, or use of antihypertensive medication; (5) FPG ≥ 100 mg/dL or use of antihyperglycemic medication.

### Statistical analysis

Statistical analyses were conducted using the SAS (version 9.3; SAS Institute, Inc., Cary, NC, USA) survey procedure and the sampling weights of KNHANES to acquire nationally representative prevalence estimates. All analyses performed in this study were adjusted for survey year to minimize the variations with survey year. The data in this study are presented as mean ± SE for continuous variables or proportion (SE) for categorical variables. General and clinical characteristics of subjects were compared according to the presence of glaucoma and obesity. Variables with skewed distributions were analyzed after logarithmic transformations. We performed a priori subgroup analysis by obesity status, which was verified by interaction between MetS and obesity (P for interaction = 0.134). Diagnoses of MetS and its components were also compared according to the presence of glaucoma and obesity status. Multiple logistic regression analysis was used to estimate the magnitude of the associations of MetS and its components with glaucoma according to obesity status after adjusting for surveyed year, age, and sex in Model 1. Model 2 additionally adjusted for IOP, household income, exercise, education level, smoking status, alcohol consumption, BMI, and dyslipidemia medication use. Odds ratios (ORs) for glaucoma according to the presence of MetS and its components were calculated separately in the non-obese and obese groups, as well as two groups together in total. Differences in prevalence of glaucoma according to number of MetS components were compared in non-obese and obese subjects. A *p-*value <0.05 was considered statistically significant.

## Additional Information

**How to cite this article**: Kim, H.-A. *et al*. Differential Association of Metabolic Risk Factors with Open Angle Glaucoma according to Obesity in a Korean Population. *Sci. Rep.* **6**, 38283; doi: 10.1038/srep38283 (2016).

**Publisher's note:** Springer Nature remains neutral with regard to jurisdictional claims in published maps and institutional affiliations.

## Figures and Tables

**Table 1:** General and Clinical Characteristics of Subjects According to the Presence of Glaucoma and Obesity.

Characteristics	Non-obese			Obese		
	Non-glaucoma (n = 5495)	Glaucoma (n = 242)	P-value	Non-glaucoma (n = 2979)	Glaucoma (n = 100)	P-value
Age (yr)	56.0 ± 0.2	61.5 ± 1.0	<0.001	55.5 ± 0.3	61.7 ± 1.5	<0.001
IOP (mmHg)	14.4 ± 0.1	15.4 ± 0.3	0.003	14.7 ± 0.1	15.5 ± 0.4	0.042
Spherical equivalent (D)	−0.5 ± 0.0	−1.4 ± 0.3	0.003	−0.7 ± 0.1	−0.7 ± 0.3	0.821
Urban (%)	77.7 (1.9)	72.2 (4.7)	0.152	75.7 (2.2)	70.0 (6.1)	0.291
Low income (%)	17.9 (0.8)	31.0 (4.3)	<0.001	18.0 (0.9)	31.6 (5.6)	0.003
Regular exercise (%)	17.7 (0.7)	13.5 (2.8)	0.190	19.0 (1.0)	19.6 (4.2)	0.871
High education (%)	58.6 (1.1)	44.0 (4.6)	0.002	55.8 (1.3)	32.9 (6.1)	<0.001
Current smoker (%)	20.6 (0.8)	25.1 (4.1)	0.244	19.9 (1.0)	16.4 (4.9)	0.517
Heavy drinker (%)	8.2 (0.5)	7.5 (2.7)	0.797	11.9 (0.8)	6.8 (2.6)	0.128
Regular physical activity (≥mod intensity, %)	19.3 (0.7)	14.8 (2.9)	0.161	19.4 (1.0)	12.8 (4.1)	0.184
Hypertension (%)	29.6 (0.8)	49.2 (4.5)	<0.001	50.0 (1.3)	69.5 (5.9)	0.003
Diabetes mellitus (%)	9.2 (0.5)	13.9 (3.6)	0.120	16.2 (0.8)	23.4 (4.8)	0.080
BMI (kg/m^2^)	22.3 ± 0.0	22.3 ± 0.2	0.914	27.3 ± 0.1	27.5 ± 0.2	0.384
WC (cm)	78.4 ± 0.1	79.7 ± 0.7	0.066	90.5 ± 0.2	91.1 ± 0.8	0.450
Systolic BP (mmHg)	120.1 ± 0.3	126.7 ± 1.7	<0.001	125.4 ± 0.4	131.9 ± 1.8	<0.001
Diastolic BP (mmHg)	74.6 ± 0.2	73.9 ± 0.8	0.365	79.2 ± 0.3	79.5 ± 1.3	0.870
FPG (mg/dL)	97.6 ± 0.4	100.5 ± 1.9	0.134	105.0 ± 0.6	107.4 ± 2.6	0.354
Total cholesterol (mg/dL)	191.8 ± 0.6	187.7 ± 3.5	0.244	199.0 ± 0.9	195.9 ± 3.5	0.413
TGs (mg/dL)	108.3 ± 2.1	129.6 ± 12.8	<0.001	143.3 ± 4.0	142.5 ± 21.2	0.940
HDL-C (mg/dL)	53.2 ± 0.2	50.0 ± 1.2	0.006	48.4 ± 0.3	50.2 ± 1.1	0.103
LDL-C (mg/dL)	113.6 ± 0.5	107.7 ± 3.0	0.054	118.1 ± 0.8	113.3 ± 3.7	0.212
MetS (%)	22.3 (0.7)	40.1 (4.6)	<0.001	58.0 (1.2)	66.0 (6.3)	0.220

Data are presented as the means ± SE, or % (SE).

Abbreviations: BMI, body mass index; WC, waist circumference; BP, blood pressure; FPG, fasting plasma glucose; TGs, triglycerides; HDL-C, high-density lipoprotein-cholesterol; LDL-C, low-density lipoprotein-cholesterol; IOP, intraocular pressure; SE, standard error.

**Table 2:** Prevalence of Metabolic Syndrome and its Components according to the Presence of Glaucoma and Obesity.

	Total			Non-obese			Obese		
	Non-glaucoma (n = 8474)	Glaucoma (n = 342)	*P*-value	Non-glaucoma (n = 5495)	Glaucoma (n = 242)	*P*-value	Non-glaucoma (n = 2979)	Glaucoma (n = 100)	*P*-value
MetS	35.2 (0.7)	47.3 (3.7)	<0.001	22.3 (0.7)	40.1 (4.6)	<0.001	58.0 (1.2)	66.0 (6.3)	0.220
High WC	39.4 (0.8)	38.3 (3.5)	0.768	18.5 (0.7)	23.3 (3.7)	0.171	76.1 (1.1)	77.2 (5.7)	0.848
High BP	47.8 (0.8)	66.2 (3.7)	<0.001	40.3 (0.9)	62.0 (4.4)	<0.001	61.1 (1.2)	76.9 (5.7)	0.017
High FPG	34.8 (0.7)	42.1 (3.7)	0.043	28.0 (0.8)	35.9 (4.7)	0.071	46.7 (1.2)	58.0 (5.6)	0.050
High TGs	38.5 (0.7)	49.3 (3.5)	0.002	30.8 (0.8)	47.4 (4.5)	<0.001	51.9 (1.2)	54.3 (6.3)	0.711
Low HDL-C	35.5 (0.7)	41.8 (3.6)	0.066	30.2 (0.8)	41.4 (4.5)	0.009	44.8 (1.1)	43.0 (5.7)	0.761

Data are presented as % (SE).

Abbreviations: WC, waist circumference; TGs, triglycerides; HDL-C, high-density lipoprotein-cholesterol; BP, blood pressure; FPG, fasting plasma glucose; MetS, metabolic syndrome; SE, standard error.

**Table 3:** Multiple Logistic Regression Analysis for the Association between Glaucoma and Metabolic Syndrome in Total population and Non-obese and Obese Participants.

	Total		Non-obese		Obese	
	Model 1	Model 2	Model 1	Model 2	Model 1	Model 2
MetS	1.37 (1.03,1.83)	1.62 (1.14,2.32)	1.96 (1.33,2.87)	2.00 (1.34,3.00)	1.05 (0.59,1.87)	1.05 (0.58,1.91)
High WC	0.91 (0.66,1.23)	1.03 (0.68,1.58)	1.36 (0.84,2.21)	1.42 (0.86,2.34)	0.77 (0.39,1.50)	0.67 (0.33,1.39)
High BP	1.58 (1.13,2.21)	1.68 (1.20,2.36)	1.89 (1.29,2.78)	1.88 (1.29,2.74)	1.37 (0.72,2.62)	1.26 (0.66,2.40)
High FPG	1.22 (0.89,1.67)	1.24 (0.87,1.76)	1.15 (0.77,1.73)	1.10 (0.72,1.68)	1.36 (0.84,2.18)	1.36 (0.84,2.22)
High TGs	1.43 (1.08,1.90)	1.41 (1.02,1.96)	1.78 (1.24,2.55)	1.65 (1.12,2.43)	1.12 (0.66,1.90)	1.02 (0.55,1.90)
Low HDL-C	1.13 (0.83,1.53)	1.08 (0.74,1.58)	1.61 (1.07,2.41)	1.37 (0.86,2.19)	0.77 (0.48,1.24)	0.62 (0.35,1.08)

Data are presented as OR (95% CI).

ORs were calculated with non-glaucoma subjects as the reference group, using multiple logistic regression analysis.

Abbreviations: WC, waist circumference; TGs, triglycerides; HDL-C, high-density lipoprotein-cholesterol; BP, blood pressure; FPG, fasting plasma glucose; MetS, metabolic syndrome; OR, odds ratio; CI, confidence interval.

^a^Model 1, adjusted for surveyed year, age and sex.

^b^Model 2, adjusted for surveyed year, age, sex, IOP, household income, exercise, education level, smoking status, alcohol consumption, and BMI (*additionally adjusted for dyslipidemia medication use).
